# Refractory Radiation Necrosis After Stereotactic Radiosurgery for Cerebellar Arteriovenous Malformation: A Case Report

**DOI:** 10.7759/cureus.85601

**Published:** 2025-06-09

**Authors:** Yurie Rai, Takahiro Ota

**Affiliations:** 1 Department of Neurosurgery, Tokyo Metropolitan Tama Medical Center, Tokyo, JPN

**Keywords:** arteriovenous malformation (avm), brain radiation necrosis, cerebellum, stereotactic radiosurgery (cyberknife®), stereotactic radiosurgery (srs)

## Abstract

Radiation necrosis is a recognized late complication of stereotactic radiosurgery for brain arteriovenous malformations, though it occurs rarely in the cerebellum. While radiation necrosis is typically managed conservatively with corticosteroids, refractory cases may require surgical intervention. A 70-year-old man underwent stereotactic radiosurgery for an incidentally detected left cerebellar arteriovenous malformation. Although complete obliteration was confirmed by digital subtraction angiography 1.5 years after stereotactic radiosurgery, he developed progressive radiation necrosis 2.5 years post-treatment, presenting with dizziness, vomiting, dysarthria, and ataxia. Initial transoral corticosteroid therapy provided symptomatic relief. Seven months later, his symptoms worsened again, and magnetic resonance imaging revealed progressive brainstem edema. Dose escalation of corticosteroids was ineffective, necessitating surgical resection. Histopathology confirmed coagulative necrosis, with remnants of the arteriovenous malformation nidus remaining. Postoperatively, the patient showed significant clinical improvement, with resolution of edema and tapering of steroids. This case highlights the challenges in managing radiation necrosis following stereotactic radiosurgery for cerebellar arteriovenous malformations. While medical therapy remains first-line, surgical resection should be considered in refractory cases to prevent complications associated with prolonged steroid use. Early recognition and intervention are crucial for optimizing patient outcomes.

## Introduction

Brain arteriovenous malformations (AVMs) carry a risk of intracranial hemorrhage, with the majority occurring in supratentorial lesions (>85%), and posterior fossa AVMs are relatively uncommon [1]. However, posterior fossa AVMs tend to be more aggressive, with an annual rupture rate of 11.6% [1]. Cerebellar AVMs are the majority of posterior fossa AVMs, while their exact frequency is unclear [2].

Stereotactic radiosurgery (SRS), such as CyberKnife, is a minimally invasive treatment option for AVMs, especially for small, deep, or complex lesions. It allows precise targeting of the nidus while sparing surrounding tissue, which is an advantage over surgical resection or endovascular embolization. However, the benefits of SRS for unruptured AVMs remain unproven, and the procedure carries the risk of late complications, including radiation-induced changes (RICs) [3]. RICs are reported to occur in approximately 30% of brain AVM patients after radiosurgery, although only 9% become symptomatic [4]. Interestingly, RICs tend to be less common in the cerebellum than in the supratentorial region [2]. Among these changes, radiation necrosis (RN) represents irreversible tissue damage and is considered one of the most severe and advanced manifestations. While RN is often manageable with medication, some cases are refractory and require surgical intervention [5,6].

To our knowledge, no prior reports have documented a case of cerebellar RN secondary to AVM treated with surgical resection and subsequent pathologic analysis. Here, we present such a rare case.

## Case presentation

**Figure 1:**
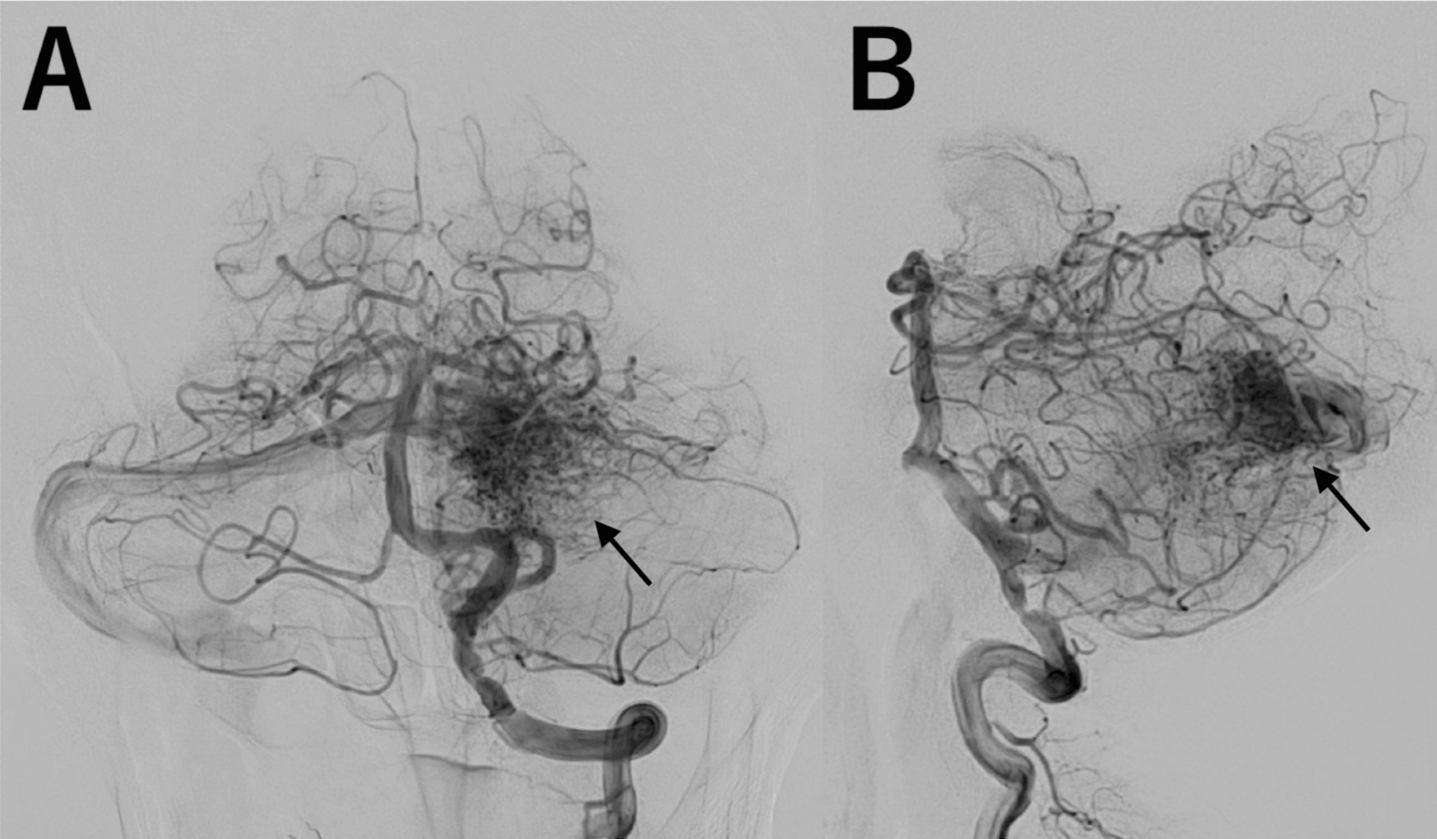
Digital subtraction angiography before stereotactic radiosurgery. A, B: Catheter angiograms of the left vertebral artery, shown in frontal view (A) and lateral view (B), demonstrating a left cerebellar arteriovenous malformation (AVM), with a nidus measuring 26 mm × 22 mm. The AVM was supplied by feeders from the bilateral superior cerebellar arteries and the left posterior inferior cerebellar artery and drained into the transverse sinus via a bridging vein.

A 70-year-old man with hypertension, dyslipidemia, and asymptomatic bilateral vertebral artery stenosis was referred for evaluation of an incidentally detected left cerebellar AVM. MRI, performed for right fingers numbness, revealed a Spetzler-Martin grade 1 AVM, later confirmed by digital subtraction angiography (DSA), with a nidus measuring 20 mm × 16 mm × 13 mm, supplied by feeders from the bilateral superior cerebellar arteries and the left posterior inferior cerebellar artery (Figures 1A, 1B, arrows). The AVM drained into the transverse sinus via a bridging vein. As the AVM was relatively small (less than 3 cm) and had numerous feeding arteries, SRS was selected as the most appropriate treatment approach rather than surgical resection or endovascular embolization. The patient subsequently underwent single-fraction 20 Gy CyberKnife SRS.

**Figure 2:**
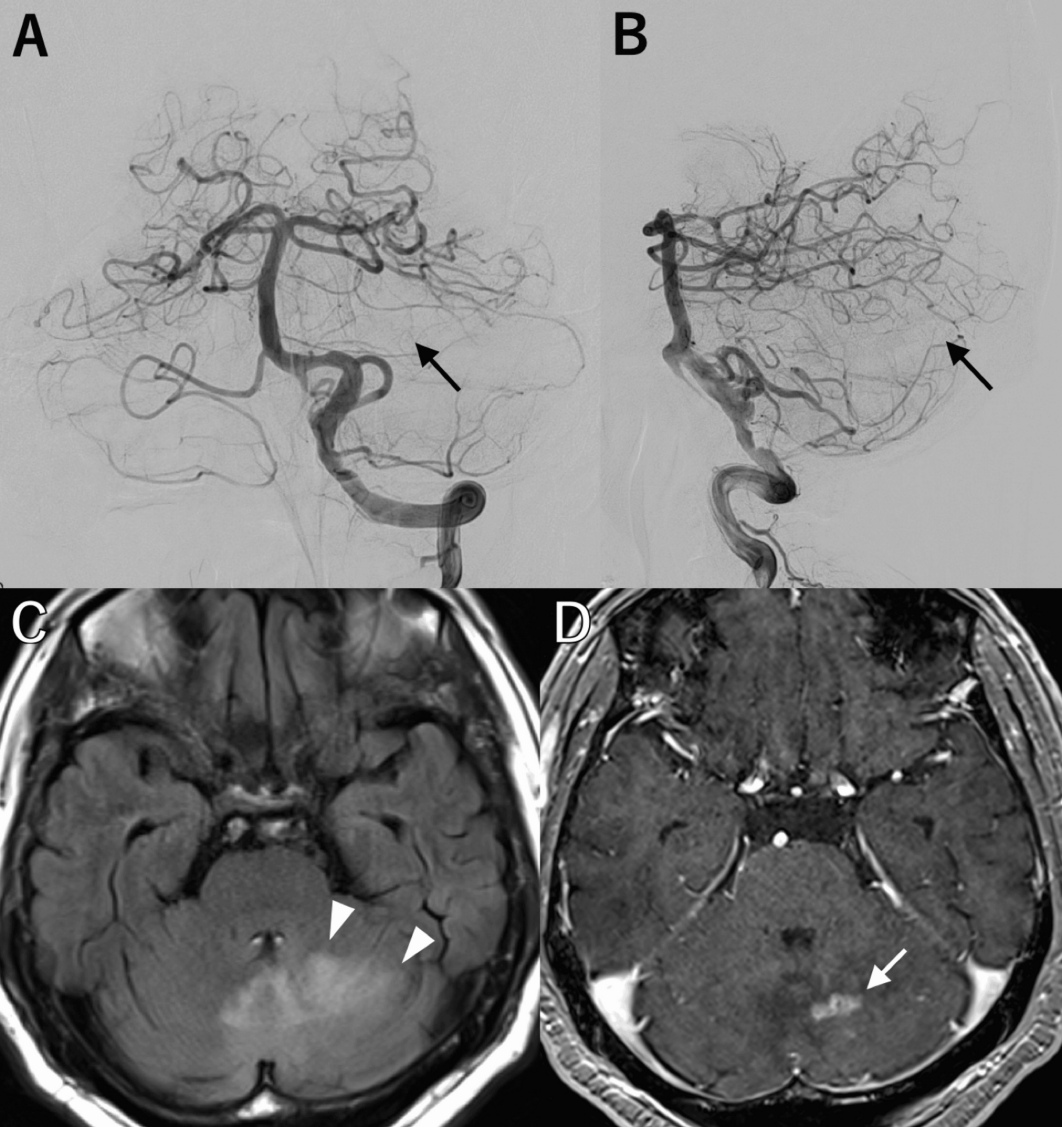
Images at six months after stereotactic radiosurgery. A, B: Catheter angiograms of the left vertebral artery, shown in frontal view (A) and lateral view (B), showing complete obliteration of the arteriovenous malformation (AVM). C, D: Magnetic resonance imaging demonstrating asymptomatic surrounding edema in the right cerebellar hemisphere on fluid-attenuated inversion recovery imaging (C, arrowheads) and a gadolinium-enhanced lesion at the prior AVM site on T1-weighted imaging (D, arrow).

Six months post-SRS, MRI showed no evidence of edema. At 1.5 years, DSA confirmed complete AVM obliteration (Figures 2A, 2B, arrows). However, at the same time, MRI revealed asymptomatic delayed RN with surrounding edema (Figure 2C, arrowheads) and a gadolinium-enhanced lesion measuring 15 mm × 13 mm × 9 mm (Figure 2D, arrow). At 2.5 years post-SRS, he developed dizziness, vomiting, mild dysarthria, and left-sided ataxia, with MRI showing progressing edema and hemorrhage. His symptoms initially improved with dexamethasone (16.5 mg), whose dose was gradually tapered to 2.5 mg over six months. During this period, he developed steroid-induced diabetes mellitus, requiring initiation of linagliptin therapy.

**Figure 3:**
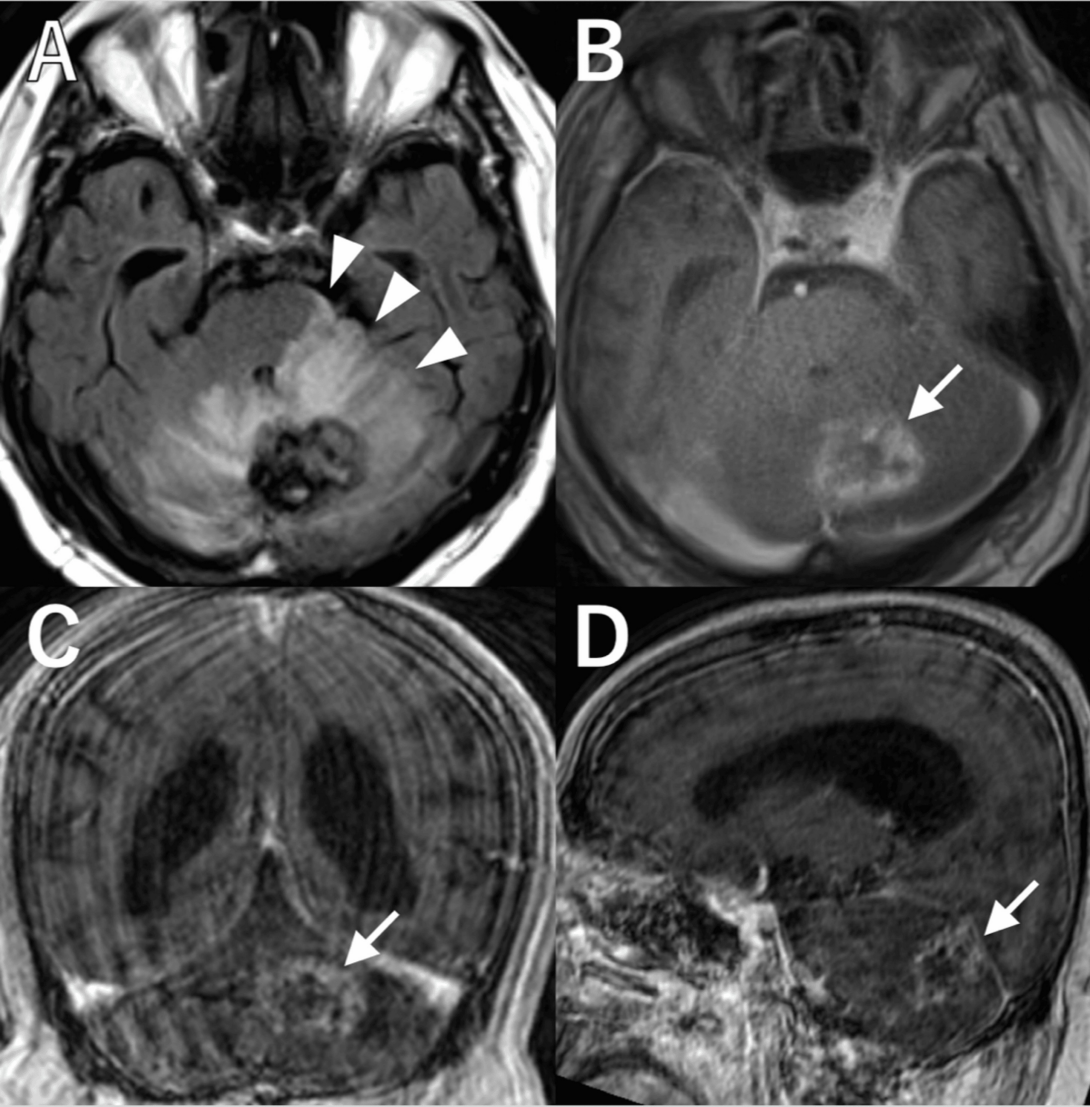
Preoperative images. A-D: Preoperative magnetic resonance imaging demonstrating significant edema in the right cerebellar hemisphere, extending to the contralateral cerebellar hemisphere and brainstem on fluid-attenuated inversion recovery imaging (A, arrowheads) and a ring-enhanced lesion at the prior arteriovenous malformation site on T1-weighted imaging with gadolinium, consistent with radiation necrosis (B: axial, C: coronal, D: sagittal, arrows).

Six months later, his ataxia worsened, and MRI showed significant worsening of edema, extending to the brainstem (Figure 3A, arrowheads). T1-weighted MRI with gadolinium enhancement revealed a progressively enlarging ring-enhanced lesion at the prior AVM site, measuring 34 mm × 25 mm × 23 mm, consistent with RN (Figures 3B-3D, arrows). Intravenous dexamethasone was increased to 12 mg, and an osmotic diuretic was added. However, his symptoms did not improve. Additionally, he developed corticosteroid-induced psychiatric disorder (CIPD) and worsening diabetes. Given that his condition was refractory to medical treatment, surgical resection of the RN was performed two weeks after admission.

**Figure 4:**
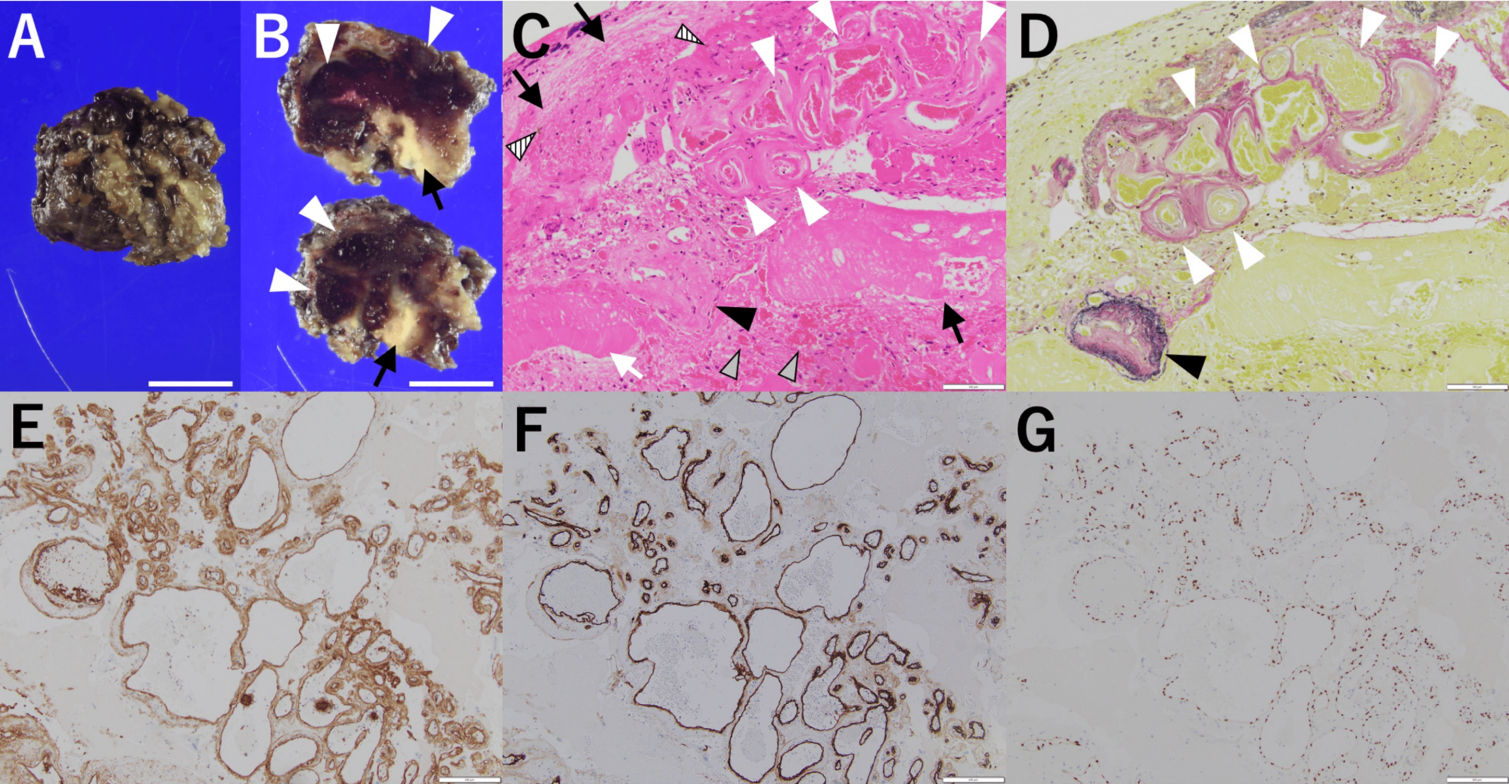
Pathological findings of the resected radiation necrosis. A, B: Macroscopic findings: Surface view (A) and the cross-sectional view (B) of the necrotic tissue, revealing numerous degenerated blood vessels (white arrowheads) surrounded by necrosis (black arrows). Scale bar: 10 mm. C, D: Microscopic findings (×200): Hematoxylin and eosin staining (C) shows coagulative necrosis (black arrows) with focal hemorrhage (gray arrowheads), hemosiderin deposition (stripe arrowheads), and irregular aggregates of abnormal arteries and veins with dilatation and thickened walls (white and black arrowheads), accompanied by focal hyalinized necrosis (white arrows). Elastin van Gieson staining (D) reveals vascular structures with (black arrowhead) and without (white arrowheads) elastic fibers. Scale bar: 100 µm. E-G: Immunohistochemical staining (×100): CD31 (E), CD34 (F) and E26 transformation-specific-related gene (ERG) (G) staining show positive endothelial expression in vascular structures. Scale bar: 200 µm.

Under general anesthesia, with the patient in the prone position, total necrotomy was conducted via a suboccipital approach. Intraoperatively, the lesion appeared solid, contained numerous degenerated blood vessels, and was surrounded by gliotic tissue. The lesion was excised en bloc, delineating the gliotic border (Figures 4A, 4B). Pathological examination revealed complete coagulative necrosis (Figure 4C, black arrows) with focal hemorrhage (Figure 4C, gray arrowheads), hemosiderin deposition (Figure 4C, stripe arrowheads), and irregular aggregates of abnormal arteries and vein with dilatation and thickened walls (Figures 4C, 4D, white and black arrowheads), accompanied by focal hyalinized necrosis (Figure 4C, white arrows). Some of these vascular structures contained elastic fibers (Figure 4D, black arrowhead) and others did not (Figure 4D, white arrowheads), consistent with AVM findings. All of these vascular structures were positive for CD31 (Figure 4E), CD34 (Figure 4F), and E26 transformation-specific-related gene (ERG) (Figure 4G) staining, indicating endothelial cells.

**Figure 5:**
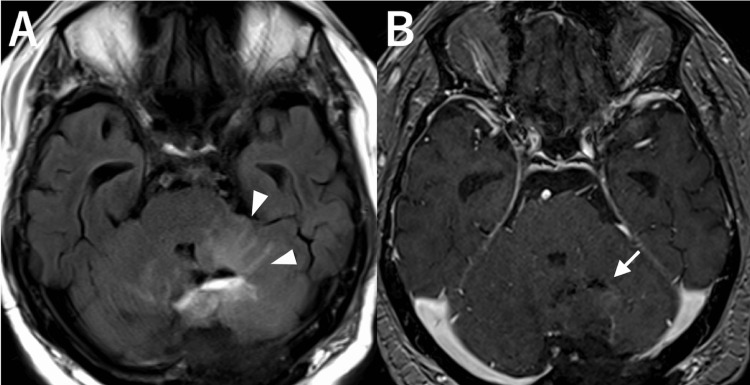
Postoperative images. A, B: Postoperative magnetic resonance imaging demonstrating significant resolution of perilesional edema on fluid-attenuated inversion recovery imaging (A, arrowheads) and complete removal of the contrast-enhanced lesion on T1-weighted imaging (B, arrow).

Postoperatively, the patient’s ataxia improved, and MRI performed two weeks after surgery demonstrated significant improvement of perilesional edema (Figure 5A, arrowheads) and complete removal of the contrast-enhanced lesion (Figure 5B, arrow). Dexamethasone was gradually tapered, and both CIPD and diabetes mellitus improved. The patient was subsequently transferred to a rehabilitation hospital.

## Discussion

RN is a well-recognized late complication of SRS for brain AVMs, though its occurrence in the cerebellum is rare [2]. In our case, we observed a progressive symptomatic cerebellar RN that was refractory to corticosteroid treatment and necessitated surgical resection. This report underscores the challenges in managing cerebellar RN and highlights the potential role of surgery as a definitive treatment in refractory cases.

Pathophysiology of RN

RN is a delayed complication of radiation therapy, characterized by irreversible tissue death due to radiation-induced vascular and cellular damage [7]. It typically occurs months to years after treatment. RICs, on the other hand, refer to a spectrum of tissue responses to radiation, including inflammation, edema, vascular remodeling, and necrosis [2,7]. These changes are usually reversible with appropriate management, except for RN, which represents the irreversible endpoint of radiation-induced damage.

Although studies specifically addressing RN of cerebellar AVMs are rare, a previous study of note compared RICs in cerebellar AVMs with those in supratentorial AVMs. The study found that RICs were less frequent in cerebellar AVMs (18.4%) than in supratentorial AVMs (37.6%). However, the rates of symptomatic and permanent RICs were comparable between the two groups [2]. This suggests that while cerebellar AVMs may have a lower overall risk of RIC, when RICs do occur, they can result in serious clinical consequences. One possible explanation is the limited space within the posterior fossa: even a small lesion can exert significant mass effect, leading to severe neurological symptoms, requiring careful management of cerebellar AVMs. At the same time, the results of this study on RICs also suggest that the pathophysiology of RIC and/or RN may differ between the cerebellum and supratentorial lesion, although the relationship between “symptomatic and permanent RICs” in this study and RN remains unclear. These differences may be attributed to structural variations between the cerebrum and cerebellum [8,9]. However, the precise mechanisms underlying these differences remain unclear.

Previous studies on RN pathophysiology propose two main hypotheses: vascular damage and glial damage. The vascular hypothesis proposes that radiation-induced endothelial damage leads to microvasculopathy, resulting in vascular insufficiency, infarction, and subsequent gray and/or white matter necrosis [10-12]. Disruption of the blood-brain barrier due to endothelial cell damage allows inflammatory cells, including T lymphocytes and macrophages, to infiltrate the perivascular space [12,13]. The glial hypothesis suggests that radiation-induced damage to glial cells leads to ablation of glial precursors, ultimately resulting in demyelination necrosis [12,14]. However, these hypotheses are primarily based on studies of cerebral or spinal RN, often in the context of tumors. As far as we searched, no reports have specifically addressed the pathophysiology of cerebellar RN following radiation treatment for AVMs. More studies are needed to better understand the mechanisms underlying these differences and their clinical implications, as well as other contributing factors such as radiation dose. Higher doses have been shown to be associated with an increased incidence of RICs and RN [15,16]. This highlights the importance of optimizing dose planning to balance efficacy and safety, particularly in eloquent or anatomically constrained regions such as the cerebellum.

Clinical presentations of RN

RN of supratentorial AVMs presents with symptoms such as headache, hemiparesis, visual disturbances, aphasia, amnesia, and epilepsy [5,17]. In contrast, RN of cerebellar AVMs manifests with symptoms including headache, dizziness, vomiting, dysarthria, and ataxia [5]. Headache is a common symptom in both groups, likely indicating increased intracranial pressure due to edema. However, other symptoms appear to differ between the two groups. The number of reported cases of cerebellar RN remains small, and this observation is still a hypothesis. Nevertheless, considering the functional specialization of different brain regions, these symptom differences appear reasonable.

Treatment strategies for RN

Medical management, primarily with corticosteroids and osmotic diuretics, is the first-line treatment for symptomatic RN [18,19]. In our case, initial steroid therapy effectively controlled the symptoms. However, the value of steroids is transient and supportive rather than curative [18]. Also, prolonged administration led to adverse effects, including steroid-induced diabetes mellitus and CIPD. This highlights the dilemma clinicians face in balancing symptom control with minimizing long-term complications of steroid therapy.

Other pharmacological options, such as bevacizumab, an anti-vascular endothelial growth factor (VEGF) agent, have been explored for refractory RN of AVMs [19,20]. However, its efficacy in cerebellar RN remains uncertain, necessitating further investigation.

Surgical considerations for refractory cases

Surgical resection of RN is not commonly performed, as most cases are effectively managed with medical therapy [6]. However, in cases where RN is refractory to medical management and results in worsening neurological symptoms, surgical intervention may be a good option. Our patient exhibited progressive ataxia, worsening brainstem edema, and complications from steroid therapy, necessitating surgical resection of the necrotic tissue. Postoperatively, the patient demonstrated significant neurological improvement, and imaging confirmed resolution of edema. The presence of gliotic tissue surrounding the necrotic core provided a clear surgical plane, allowing safe en bloc resection. These findings suggest that in select cases, surgical excision can be a viable and effective treatment strategy for relieving mass effect caused by necrosis.

Clinical implications and future directions

This case emphasizes the importance of early recognition and intervention in symptomatic cerebellar RN following SRS. Given the higher risk of mass effect in the posterior fossa, close post-SRS monitoring with MRI is essential for early detection of edema and necrosis. In cases where corticosteroids are ineffective or poorly tolerated, surgical resection should be considered promptly. Future studies are needed to establish standardized treatment protocols and to evaluate alternative pharmacological therapies that may mitigate the need for surgical intervention. Additionally, a deeper understanding of the pathophysiology of cerebellar RN is crucial for developing targeted therapeutic strategies. As post-SRS necrosis in cerebellar AVMs is rarely reported, accumulating more cases and long-term follow-up data will be essential for a better understanding of its clinical course and optimal management.

## Conclusions

This report presents a rare case of refractory radiation necrosis in the cerebellum following stereotactic radiosurgery for AVM, successfully treated with surgical resection. Histopathological examination revealed remnants of abnormal vascular structures within necrotic brain tissue. Although SRS is generally safe and effective for cerebellar AVMs, clinicians should be aware of the potential for delayed RN. In cases unresponsive to corticosteroids or complicated by adverse effects, early surgical intervention should be considered.
